# Antimicrobial resistance and a diminishing pool of reserved antibiotics

**DOI:** 10.1590/1516-3180.2019.0368120619

**Published:** 2019-09-23

**Authors:** Ihsan Ullah Khan Altaf, Ahmad Khan, Amjad Mahboob

**Affiliations:** I PharmD, MPhil. Doctoral Student, Department of Pharmacy, Quaid-i-Azam University, Islamabad, Pakistan. Pharmacist, Bacha Khan Medical Complex, Swabi, Khyber Pakhtunkhwa, Pakistan.; II BPharm, MPhil, PhD. Assistant Professor, Department of Pharmacy, Quaid-i-Azam University, Islamabad, Pakistan.; III MBBS, FCPS (Medicine), FCPS (Infectious Diseases). Assistant Professor and Infectious Disease Specialist, Bacha Khan Medical Complex, Swabi, Khyber Pakhtunkhwa, Pakistan.

Dear Editor,

During the second half of the nineteenth century, infectious diseases were a prominent cause of morbidity and mortality. Almost one-third of the infants in the United States and Western Europe were unable to reach their first birthday due to causes attributable to infectious diseases.^1^

Antibiotics have played a significant role in saving patients’ lives through prevention and treatment of complicated infections, such as their use after the surgical procedures of joint replacement, organ transplantation or cardiac surgery. In fact, in areas of poor sanitation, antibiotics have also helped in lowering the morbidity and mortality associated with food-borne diseases.

However, through overuse of antibiotics, inappropriate use, self-medication, lack of adherence and inappropriate prescribing, the phenomenon of antibiotic resistance has rapidly spread from healthcare settings to the community worldwide.^2^ With the passage of time, the concept of multidrug-resistant (MDR) bacterial strains has emerged and, as a result, antibiotics have lost their efficacy. This has further progressed towards extended hospitalization, complications, massive costs and an upswing in morbidity and mortality.^3^

Currently, antimicrobial resistance (AMR) is one of the major public health challenges across the globe. If the process of drug resistance does not become controlled smartly, it is estimated that mortality attributable to antimicrobial resistance will exceed 10 million deaths annually by 2050, compared with current estimates of 0.7 million deaths. In other words, one person will die every three seconds due to a resistant organism. Excessive and irrational use of antibiotics in the community and in healthcare settings is one of the key factors that promote drug resistance.^4^

Entry to the post-antibiotic era, i.e. the time when most antibiotics will have become ineffective due to development of resistance against them, requires serious measures with the capacity to tackle this global issue in the form of collaborative effort. The Lancet Infectious Diseases Commission and the World Health Organization (WHO) have emphasized the need for a common approach to help developing countries improve their use of antibiotics through stewardship and education, and also through the requirement to develop national action plans for antimicrobial resistance. The Antimicrobial Stewardship Program (ASP) has been described by the Society of Healthcare Epidemiology of America and the Infectious Diseases Society of America as “a multidisciplinary approach by a team consisting of infectious disease clinicians, pharmacists, microbiologists, hospital epidemiologists and infection preventionists”.^5^ This stewardship can be divided into core and additional elements. Its core elements comprise various activities such as pre-authorization and formulary restriction, feedback and prospective audit, whereas its additional elements include development of evidence-based guidelines, education, antimicrobial ordering forms, optimization of doses by means of de-escalation and parenteral-to-oral conversions of antimicrobials (where required).

For limited-resource settings, the easiest way to start containment of antimicrobial resistance through the Antimicrobial Stewardship Program is to conduct surveillance of antibiotic use that initially targets the WATCH group (antibiotics that are recommended for specific indications and are at greater risk of becoming ineffective due to development of resistance against them) and the RESERVE group (antibiotics that are lifesaving last lines of defense, which include medicines like intravenous fosfomycin and colistin). This can be followed by interventions to contain any inappropriate use through education, restrictions and raised public health awareness.

Thus, through following these steps, the diminishing pool of reserve antibiotics can be safeguarded for the coming generations around the world.
